# “3D” Techniques for Improving Safety in Posterior Knee Arthroscopy During Posterior Cruciate Ligament Avulsion Fracture Fixation: A Series of Key Technical Modifications

**DOI:** 10.1002/atn2.70340

**Published:** 2026-10-09

**Authors:** Zhichen Zou, Xinbei Dong, Chuanzheng Zhu, Qinglong Wang, Zhixue Wang

**Affiliations:** ^1^ Department of Orthopedics, Tangdu Hospital Air Force Medical University Xi'an Shaanxi China

## Abstract

Arthroscopic procedures in the posterior compartment of the knee are technically demanding and present an inherent risk of iatrogenic neurovascular injury because of the close proximity of the popliteal neurovascular bundle. This technical note aims to enhance surgical safety by describing a series of modifications termed the “3D” technique. The technique integrates 3 simple, practical, and reproducible modifications that improve visualization and tunnel accuracy while minimizing the risk of complications using only standard arthroscopic instruments.

## Graphical abstract

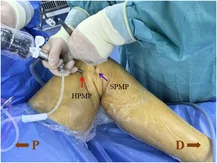

**VIDEO 1:** “3D” techniques for improving safety in posterior knee arthroscopy during posterior cruciate ligament avulsion fracture fixation: a series of key technical modifications. The authors declare that they have no competing financial interests or personal relations that could have influenced the work reported in this video. The technique we describe is applicable to posterior cruciate ligament (PCL) reconstruction or reduction and fixation of PCL tibial avulsion fractures. 3D techniques represent the following: first D, distalization of the neurovascular bundle; the second D, dual posteromedial portal; the third D, depth‐limited drilling. This is a patient with a PCL tibial avulsion fracture. We position the affected leg in a figure‐of‐4 posture with the knee hyperflexed. At the same time, we adjust the irrigation pressure to keep the posterior capsule distended. This helps move the neurovascular bundle away from our working area. We advance the arthroscope into the posteromedial compartment and establish a standard posteromedial portal. Under spinal needle guidance, we create a high posteromedial portal. As shown in the image, the spinal needle indicates the high posteromedial portal, and the switching stick indicates the standard posteromedial portal. We then switch the arthroscope to the high posteromedial portal. From this view, we can clearly observe the posterior compartment of the knee, including the posterior aspects of the lateral and medial femoral condyles, as well as the tibial attachment of the PCL. Expose the fracture fragment and mark the inferior margin of the bone bed. Then, prepare for tunnel creation. Before creating the tunnel, we place an outside‐in guide through the standard posteromedial portal and position it at the intended tunnel aperture. The sleeve is advanced until it contacts the anterior tibial cortex. Then, we measure and record the remaining length of the sleeve. After that, we withdraw the guide from the joint and reproduce the measured length outside the joint. The guide pin is then inserted into the sleeve and secured such that its length slightly exceeds the measured depth. With the depth limit in place, as soon as the guide pin tip passes through the cortex, we can observe it immediately, and the pin stops advancing on its own. We use the same method to create the second tunnel. This depth‐limited drilling technique helps avoid overpenetration and reduces the risk of neurovascular injury. Thanks for your attention! Video content can be viewed at https://doi.org/10.1002/atn2.70340.

Although knee arthroscopy has become a well‐established and widely performed technique, visualization and instrumentation of the posterior compartment remain challenging,^1^ particularly during posterior cruciate ligament (PCL) reconstruction or reduction and fixation of PCL avulsion fractures. Neurovascular injury represents the most devastating complication of these procedures, with an incidence rate of up to 9% reported in the literature.^2^, ^3^ Therefore, minimizing the risk of iatrogenic neurovascular injury while maximizing surgical safety during arthroscopic procedures in the posterior compartment remains one of the foremost concerns for surgeons. To address this concern, we describe a series of technical modifications to enhance surgical safety, summarized as the “3D” techniques (with each modification beginning with the letter “D”).

## SURGICAL TECHNIQUE

### Patient Positioning and Conventional Procedures

After successful anesthesia induction, the patient is positioned supine on the operating table. A pneumatic tourniquet is applied to the proximal thigh of the affected limb. After standard sterilization and draping, conventional anterolateral and anteromedial knee portals are established. First, intra‐articular debridement is performed, and concomitant injuries, such as meniscal tears, are addressed accordingly. Subsequently, the 3 key modifications of the “3D” techniques are implemented as described below.

### First D: Distalization of the Neurovascular Bundle

Two technical modifications are implemented to maximize the distance from the neurovascular bundle, thereby minimizing the risk of iatrogenic injury. The first modification involves placing the affected lower extremity in a figure‐of‐4 position and hyperflexing it to >100° (Figure 1). Anatomical studies have confirmed that hyperflexion of the knee maximizes the distance between the neurovascular bundle and the posterior compartment, thus optimizing surgical safety.^4^, ^5^, ^6^ Additionally, throughout the procedure, the irrigation pressure is appropriately elevated and maintained to achieve sustained capsular distention. This maneuver confers a dual benefit: it displaces the neurovascular bundle further posteriorly, thereby increasing the safety margin while simultaneously expanding the working space within the posterior compartment to optimize visualization. Increased irrigation pressure can be achieved either by elevating the saline bag (gravity‐fed system) or by adjusting the pump pressure settings (automated pump system).^7^

**FIGURE 1:**
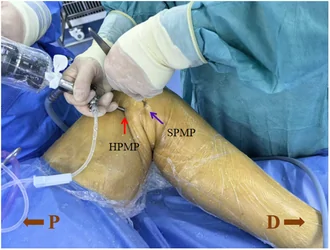
Arthroscopic instrumentation of the posterior compartment using the 3D technique (left knee). The affected lower extremity is positioned in a figure‐of‐4 posture with the knee hyperflexed to ≥100°. Dual posteromedial portals are established: the high posteromedial portal serves as the viewing portal (red arrow), and the standard posteromedial portal functions as the working portal (purple arrow). (D, distal; HPMP, high posteromedial portal; P, proximal; SPMP, standard posteromedial portal.)

### Second D: Dual Posteromedial Portal

The arthroscope (Stryker, Kalamazoo, MI) is introduced into the posteromedial compartment between the PCL and the medial femoral condyle via the transnotch approach. Under direct arthroscopic visualization, a standard posteromedial portal is established. Approximately 4 to 5 cm proximal to the standard posteromedial portal, a high posteromedial portal is established under spinal needle guidance (Figure 1). Typically, the high posteromedial portal serves as the viewing portal, whereas the standard posteromedial portal functions as the working portal (Figure 2). Compared with visualization achieved through the anterolateral or anteromedial portals, this dual posteromedial portal setup optimizes visualization and facilitates thorough debridement and exposure of the tibial attachment of the PCL under direct arthroscopic visualization. For PCL avulsion fractures, this approach also enables the distal extension of capsular dissection beyond the avulsion fragment, allowing adequate exposure of its inferior margin. The exposure achieved is sufficient to prepare for subsequent tunnel creation. The dual posteromedial portal setup enables surgical instrumentation of the posterior compartment under direct visualization, thereby reducing the risk of posterior capsular violation and iatrogenic neurovascular injury, thus enhancing overall surgical safety.^8^ Furthermore, this setup facilitates accurate localization of the tibial tunnel and minimizes coronal‐plane deviation of the tunnel aperture.

**FIGURE 2:**
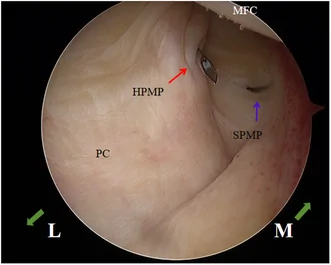
Arthroscopic view of the dual posteromedial portals. The arthroscope is introduced into the posteromedial compartment between the posterior cruciate ligament and the medial femoral condyle. The standard posteromedial portal (purple arrow) and high posteromedial portal (red arrow) are established under spinal needle guidance. (HPMP, high posteromedial portal; L, lateral; M, medial; MFC, medial femur condylar; PC, posterior capsule; SPMP, standard posteromedial portal.)

### Third D: Depth‐Limited Drilling

Both PCL reconstruction and arthroscopic fixation of PCL avulsion fractures require the creation of a tibial tunnel. The depth‐limited drilling technique involves positioning the bow‐shaped drill guide (Smith & Nephew, Memphis, TN) at the intended tibial tunnel site before tunnel creation, with the guide sleeve advanced until it contacts the anterior tibial cortex. The remaining length of the sleeve from its junction with the bow‐shaped guide to its free end is then measured and recorded to determine the drilling depth (Figure 3A). The drill guide is then withdrawn from the joint, and the previously measured remaining length of the sleeve is reproduced extracorporeally (Figure 3B,C). The guide pin is then inserted into the sleeve and secured such that its length slightly exceeds the measured depth, thereby allowing adequate penetration without risking overpenetration (Figure 3D). Tibial tunnel drilling is then performed. Under direct arthroscopic visualization, the guide pin exactly penetrates the posterior cortex and simultaneously reaches the preset depth limit (Figure 4A,B), thereby preventing overpenetration that could cause capsular violation and neurovascular injury. This depth‐limiting technique prevents overpenetration of the guide pin during tunnel drilling, thereby avoiding iatrogenic injury to the popliteal neurovascular bundle. For the detailed procedure, refer to Video 1.

**FIGURE 3:**
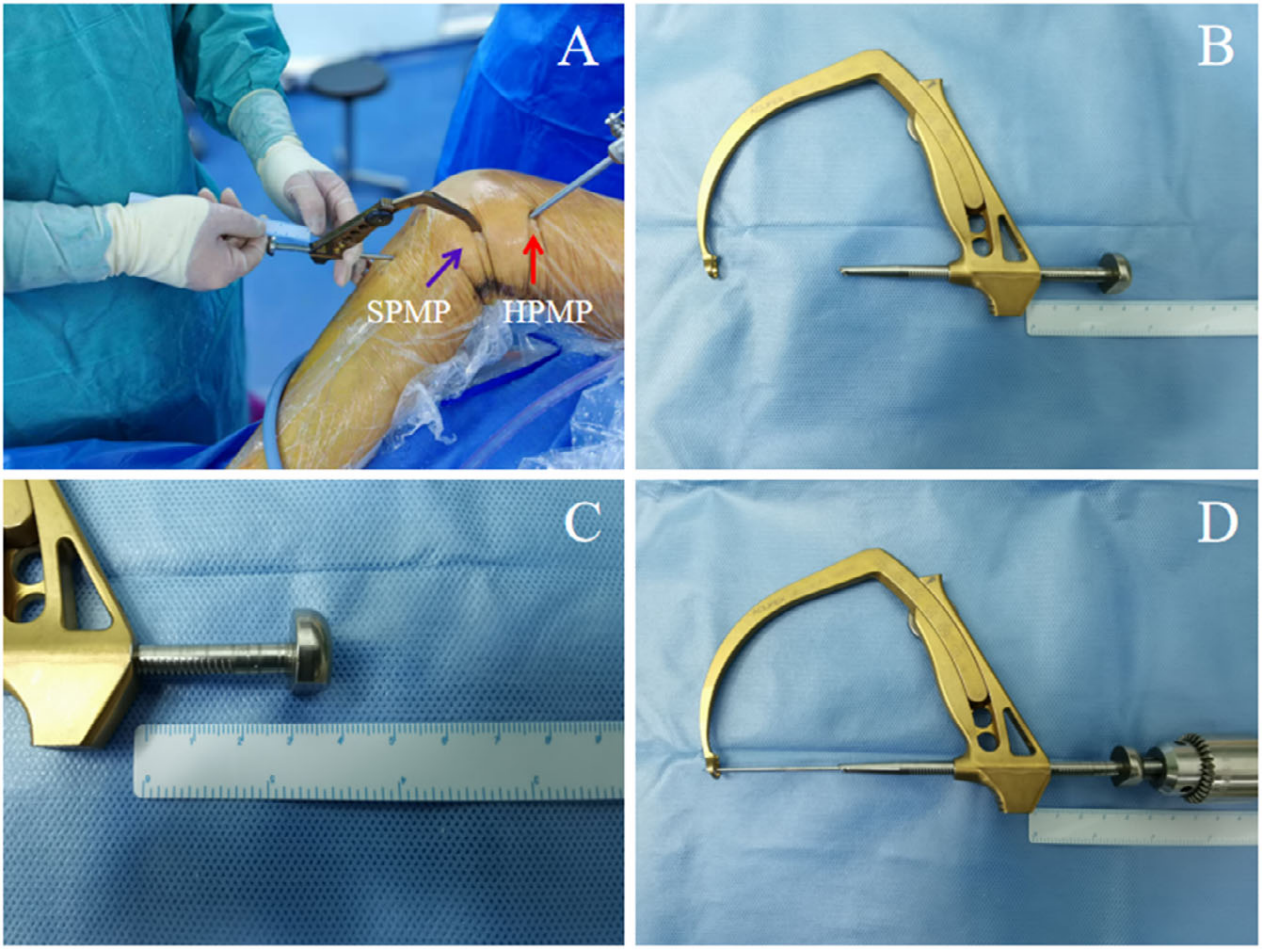
Depth‐limited drilling. (A) The arthroscope is introduced into the posterior compartment through the high posteromedial portal (red arrow). Under direct arthroscopic visualization, a bow‐shaped drill guide (outside‐in guide) is introduced through the standard posteromedial portal (purple arrow) and positioned at the intended tunnel aperture. The guide sleeve is advanced until it contacts the anterior tibial cortex, and the remaining length of the sleeve is measured and recorded. (B,C) The drill guide is then withdrawn from the joint, and the previously measured remaining length of the sleeve is reproduced extracorporeally. (D) The guide pin is then inserted into the sleeve and secured such that its length slightly exceeds the measured depth. (HPMP, high posteromedial portal; SPMP, standard posteromedial portal.)

**FIGURE 4:**
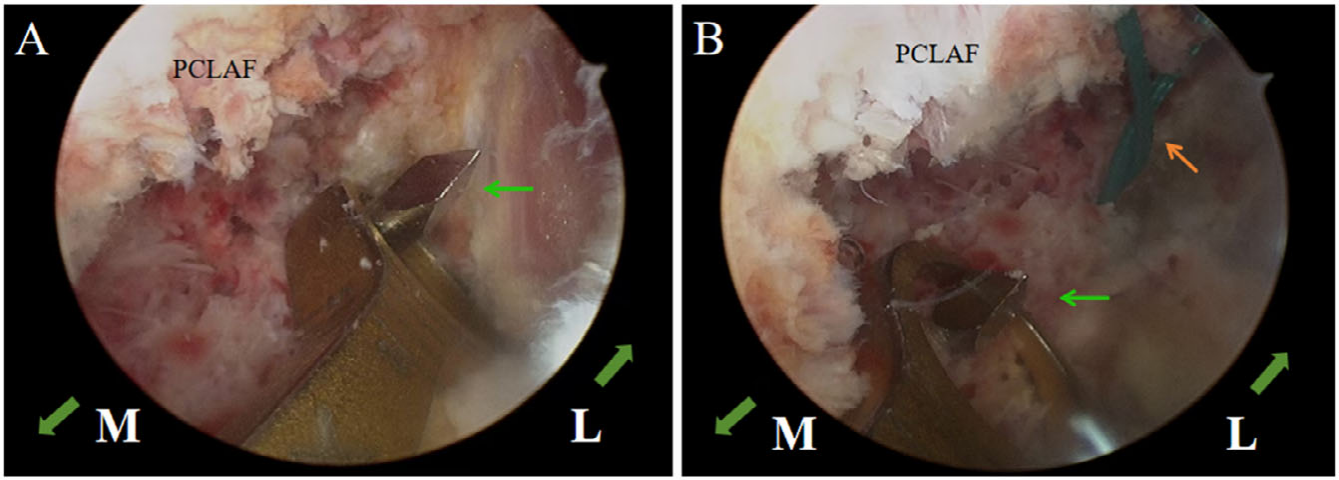
Application of the 3D techniques for reduction and fixation of a posterior cruciate ligament tibial avulsion fracture (left knee). (A) After depth‐limited setting of the guide pin, tunnel creation is performed at the inferior margin of the bone bed of the avulsion fragment. The guide pin tip (green arrow) precisely penetrates the posterior cortex and simultaneously reaches the preset depth limit, at which point advancement ceases. (B) After tunnel creation, the reinforcing suture (orange arrow) is introduced. A second tunnel is created using the same method. Under depth‐limited setting and direct arthroscopic visualization, the guide pin tip (green arrow), on penetrating the posterior cortex, appears at the ring target of the guide and can be immediately observed. (L, lateral; M, medial; PCLAF, posterior cruciate ligament avulsion fracture.)

## DISCUSSION

The reduction and fixation techniques for PCL avulsion fractures have been extensively described in the literature.^9^, ^10^, ^11^ However, arthroscopic procedures in the posterior compartment of the knee present unique challenges owing to limited visualization and the close anatomical proximity of the popliteal neurovascular bundle. Although knee arthroscopy is generally considered a safe procedure, complications are not negligible. In a large‐scale analysis of 138,823 arthroscopic knee procedures, the overall complication rate was 7.53%, with PCL reconstruction carrying the highest rate at 26.38%.^12^ Similarly, arthroscopic management of PCL tibial avulsion fractures poses comparable technical challenges, with a reported complication rate of 12.5% in a recent systematic review.^13^ These findings emphasize the need for practical, reproducible safety modifications.

To address these challenges, we describe a series of technical modifications, each beginning with the letter “D.” This simple, mnemonic summary is intended to reduce the complication rate and enhance surgical safety. The 3D technique provides a comprehensive, step‐by‐step description encompassing patient positioning, portal establishment, and the technical details of tunnel drilling throughout the entire procedure. The first D enhances surgical safety through 2 simple modifications: increasing knee flexion and maintaining appropriate capsular distention. The second and third Ds—establishment of dual posteromedial portals and implementation of depth‐limited drilling—may slightly prolong the operative time; however, they significantly improve visualization of the posterior compartment and tunnel accuracy while reducing the risk of neurovascular injury. Table 1 summarizes the advantages and limitations of our technique, whereas Table 2 outlines its key pearls and pitfalls.

**TABLE 1:** Advantages and Limitations

**Advantages**
Enhanced safety: hyperflexion and capsular distention displace the neurovascular bundle, increasing the margin of safety and reducing the risk of iatrogenic injury to the popliteal vessels
Improved visualization: the dual posteromedial portal setup provides independent viewing and working channels, optimizing exposure of the posterior compartment, particularly the PCL tibial attachment
The depth‐limited drilling technique prevents guide pin overpenetration, ensuring precise tunnel creation and minimizing coronal‐plane deviation, thereby reducing the risk of iatrogenic neurovascular injury
Reproducibility: all 3 modifications use standard arthroscopic instruments and do not require specialized or costly disposable devices, making the technique easily reproducible

**Limitations**
Learning curve: the dual posteromedial portal setup requires familiarization with the specific portal placement (4‐5 cm apart) and triangulation techniques
Potential for increased operative time: the establishment of dual portals and the application of the depth‐limited drilling technique may slightly prolong the initial operative time compared with standard approaches
Patient‐related restrictions: the technique requires adequate preoperative knee flexion (>100°). Patients with pre‐existing stiffness may not be suitable candidates

PCL, posterior cruciate ligament.

**TABLE 2:** Pearls and Pitfalls

**Pearls**
The figure‐of‐4 hyperflexed position of the knee must be maintained throughout the procedure to prevent loss of flexion angle caused by slippage on the smooth sterile drapes
Recommend using a ring guide (outside‐in guide) introduced through the standard posteromedial portal into the posterior compartment for tibial tunnel creation. The advantage of this approach is that it allows the surgeon to visualize the guide pin tip immediately on penetration of the posterior cortex, thereby enhancing safety

**Pitfalls**
A sufficient distance must be maintained between the dual posteromedial portals, typically 4‐5 cm, to prevent the 2 portals from being too close and interfering with each other during instrumentation
Violation of the posterior knee capsule or prolonged operative time may allow continuous extravasation of irrigation fluid into the soft‐tissue compartments of the calf, leading to severe postoperative swelling

In summary, the “3D” technique integrates 3 simple, practical, and reproducible modifications—distalization of the neurovascular bundle, dual posteromedial portals, and depth‐limited drilling—to enhance safety during posterior knee arthroscopy. This approach improves visualization and tunnel accuracy while minimizing the neurovascular risk.

## DISCLOSURES

The authors (Z.Z., X.D., C.Z., Q.W., Z.W.) declare that they have no known competing financial interests or personal relationships that could have appeared to influence the work reported in this paper.

## References

[1] Greenwood K , Van Zyl R , Keough N , Hohmann E . The determination of safe zones for arthroscopic portal placement into the posterior knee by mapping the courses of neurovascular structures in relation to bony landmarks. Eur J Orthop Surg Traumatol. 2021;31:1087‐1095. 10.1007/s00590-020-02847-4 33389054

[2] Zsidai B , Horvath A , Winkler PW , et al. Different injury patterns exist among patients undergoing operative treatment of isolated PCL, combined PCL/ACL, and isolated ACL injuries: A study from the Swedish National Knee Ligament Registry. Knee Surg Sports Traumatol Arthrosc. 2022;30:3451‐3460. 10.1007/s00167-022-06948-x PMC9464165 35357530

[3] Strydom A , van Wyk J , Scheffer C , Keough N . A cadaveric study to test the viability of pre‐determined safe zones for arthroscopic portal placement into the posterior knee. Arthroscopy. 2023;39:1489‐1498.

[4] Mizuno T , Hasegawa H , Tsukada T , et al. Deep flexion helps to avoid popliteal artery injury during all‐inside lateral meniscal repair: A cadaveric study. Knee. 2021;33:159‐168. 10.1016/j.knee.2021.09.004 34624750

[5] Hungerford M , Dunbar N , Varner T , et al. Measured distance from posterior tibia to popliteal artery increases with flexion and subluxation of the knee. J Exp Orthop. 2024;11:e12070. 10.1002/jeo2.12070 PMC11217668 38957228

[6] Cuéllar A , Cuéllar R , Cuéllar A , Garcia‐Alonso I , Ruiz‐Ibán MA . The effect of knee flexion angle on the neurovascular safety of all‐inside lateral meniscus repair: A cadaveric study. Arthroscopy. 2015;31:2138‐2144. 10.1016/j.arthro.2015.04.100 26163307

[7] Walker‐Santiago R , Maldonado DR , Domb BG , Lall AC . Fundamentals of arthroscopy fluid management and strategies to safely improve visualization. J Am Acad Orthop Surg. 2021;29:862‐871. 10.5435/JAAOS-D-20-01057 34623341

[8] McGinnis MD IV , Gonzalez R , Nyland J , Caborn DNM . The posteromedial knee arthroscopy portal: A cadaveric study defining a safety zone for portal placement. Arthroscopy. 2011;27:1090‐1095. 10.1016/j.arthro.2011.02.031 21620634

[9] Thonse C , Magar MT , Paul D . Arthroscopic “Crisscross” fixation technique for avulsion fracture of the posterior cruciate ligament from the Tibia. Arthrosc Tech. 2024;14:103277. 10.1016/j.eats.2024.103277 PMC11977143 40207346

[10] Capella M , D’Antonio D , Drocco L , et al. Arthroscopic all‐inside posterior cruciate ligament avulsion fracture suture fixation with double‐tunnel pullout and high‐strength suture tape augmentation using trans‐septal approach. Arthrosc Tech. 2024;13:103091. 10.1016/j.eats.2024.103091 PMC11520033 39479023

[11] Wu S , Zhang R , Zheng Y , et al. Suture bridge technique for arthroscopic repair of posterior cruciate ligament tibial avulsion. Arthrosc Tech. 2025;14:103558. 10.1016/j.eats.2025.103558 PMC12350228 40822181

[12] Bragg JT , McIntyre JA , Puzzitiello RN , Salzler MJ . Complications, reoperations, and readmissions after common arthroscopic sports medicine procedures of the knee: An analysis of the ABOS part II oral examination case list database. Am J Sports Med. 2025;53:1085‐1092. 10.1177/03635465251321007 39989089

[13] Hooper PO 3rd , Silko C , Malcolm TL , Farrow LD . Management of posterior cruciate ligament tibial avulsion injuries: A systematic review. Am J Sports Med. 2018;46:734‐742. 10.1177/0363546517701911 28437619

